# Childhood Trauma in Schizophrenia: Current Findings and Research Perspectives

**DOI:** 10.3389/fnins.2019.00274

**Published:** 2019-03-21

**Authors:** David Popovic, Andrea Schmitt, Lalit Kaurani, Fanny Senner, Sergi Papiol, Berend Malchow, Andre Fischer, Thomas G. Schulze, Nikolaos Koutsouleris, Peter Falkai

**Affiliations:** ^1^Department of Psychiatry and Psychotherapy, University Hospital, Ludwig Maximilian University of Munich, Munich, Germany; ^2^International Max Planck Research School for Translational Psychiatry (IMPRS-TP), Munich, Germany; ^3^Laboratory of Neuroscience (LIM27), Institute of Psychiatry, University of São Paulo, São Paulo, Brazil; ^4^German Center of Neurodegenerative Diseases, University of Göttingen, Göttingen, Germany; ^5^Institute of Psychiatric Phenomics and Genomics, University Hospital, Ludwig Maximilian University of Munich, Munich, Germany; ^6^Department of Psychiatry and Psychotherapy, University Hospital of Jena, Jena, Germany

**Keywords:** neurodevelopment, childhood trauma, diagnostic tools, schizophrenia, machine learning

## Abstract

Schizophrenia is a severe neuropsychiatric disorder with persistence of symptoms throughout adult life in most of the affected patients. This unfavorable course is associated with multiple episodes and residual symptoms, mainly negative symptoms and cognitive deficits. The neural diathesis-stress model proposes that psychosocial stress acts on a pre-existing vulnerability and thus triggers the symptoms of schizophrenia. Childhood trauma is a severe form of stress that renders individuals more vulnerable to developing schizophrenia; neurobiological effects of such trauma on the endocrine system and epigenetic mechanisms are discussed. Childhood trauma is associated with impaired working memory, executive function, verbal learning, and attention in schizophrenia patients, including those at ultra-high risk to develop psychosis. In these patients, higher levels of childhood trauma were correlated with higher levels of attenuated positive symptoms, general symptoms, and depressive symptoms; lower levels of global functioning; and poorer cognitive performance in visual episodic memory end executive functions. In this review, we discuss effects of specific gene variants that interact with childhood trauma in patients with schizophrenia and describe new findings on the brain structural and functional level. Additive effects between childhood trauma and brain-derived neurotrophic factor methionine carriers on volume loss of the hippocampal subregions cornu ammonis (CA)4/dentate gyrus and CA2/3 have been reported in schizophrenia patients. A functional magnetic resonance imaging study showed that childhood trauma exposure resulted in aberrant function of parietal areas involved in working memory and of visual cortical areas involved in attention. In a theory of mind task reflecting social cognition, childhood trauma was associated with activation of the posterior cingulate gyrus, precuneus, and dorsomedial prefrontal cortex in patients with schizophrenia. In addition, decreased connectivity was shown between the posterior cingulate/precuneus region and the amygdala in patients with high levels of physical neglect and sexual abuse during childhood, suggesting that disturbances in specific brain networks underlie cognitive abilities. Finally, we discuss some of the questionnaires that are commonly used to assess childhood trauma and outline possibilities to use recent biostatistical methods, such as machine learning, to analyze the resulting datasets.

## Introduction

Schizophrenia is a severe neuropsychiatric disorder that affects about 1% of the population (Jablensky, 1995). It is particularly prevalent in young adults between 20 and 30 years of age and leads to disability in about half of the patients (Murray and Lopez, 1996). The disorder is among the leading cause of years lived with disability worldwide (Whiteford et al., 2013), and, among all mental illnesses, schizophrenia is associated with the highest socioeconomic costs (Gustavsson et al., 2011). This high disorder burden is due to the early onset of schizophrenia in late adolescence and early adulthood and the persistence of symptoms throughout adult life in over 90% of affected patients despite meeting remission criteria (Häfner and an der Heiden, 2007; Schennach et al., 2015). Symptom improvement has been measured as “therapeutic response,” which was defined by, e.g., a 20% symptom reduction after 4 weeks of treatment (Kane et al., 1984). Subsequently, the term “remission” was introduced, requiring a simultaneous reduction of all diagnosis-specific core symptoms (positive and negative symptoms) to a level of “mild or less” on established questionnaires (Positive and Negative Syndrome Scale, Brief Psychiatric Rating Scale, Scale for the Assessment of Positive Symptoms, Scale for the Assessment of Negative Symptoms) for a minimum of 6 months (Andreasen et al., 2005). However, since only a small portion of schizophrenia patients can achieve this, the new definition of “recovery” was conceived, which takes into account not only a reduction of clinical symptoms, but also an improvement in occupational, social and adaptive functioning (Chan et al., 2018). However, only 20% of people with schizophrenia are able to work in the primary labor market, and only about 30% are able to maintain a stable relationship (Häfner and an der Heiden, 2007). The unfavorable disorder course is associated with multiple episodes and residual symptoms, mainly negative symptoms and cognitive deficits (McGrath et al., 2008). Cognitive deficits as a core feature of the disorder are present in domains such as episodic memory, executive function, social cognition, and attention (Green, 1996; Hoff et al., 2005). These deficits may lead to memory decline, social withdrawal, and ultimately impaired social and role functioning as measured by the Global Assessment of Functioning (GAF) scale (Green et al., 2015a). Several studies have operationalized the term “recovery” by using the GAF scale to investigate the long-term outcome and its relevant influencing factors in psychosis patients (Scott et al., 2013; Amminger et al., 2015; Koutsouleris et al., 2016; Jagannath et al., 2018; Lho et al., 2019). While childhood trauma has been repeatedly shown to negatively impact “recovery” among schizophrenia patients (Alameda et al., 2015, 2017; Trauelsen et al., 2016), some of these findings were only partially replicated (Trotta et al., 2016; Ajnakina et al., 2018), hereby leading to a rather heterogeneous body of evidence and consequently emphasizing the need for further research into the neurobiological underpinnings of this association.

## Risk Factors for Schizophrenia and the Neurodevelopmental Hypothesis

Twin studies found a heritability of about 60–80% for schizophrenia (Sullivan et al., 2003), and new genome-wide association studies (GWASs) revealed a total of 145 genetic risk loci, the single nucleotide polymorphisms (SNPs), each with only a weak effect (Pardinas et al., 2018). GWAS-based schizophrenia polygenic risk scores showed associations with social and cognitive impairments during early childhood, which were interpreted as being possible early manifestations of genetic liability (Riglin et al., 2017). In schizophrenia, however, about 8,300 SNPs have been estimated to contribute to a common risk of only 32% (Ripke et al., 2013), suggesting that—in addition to the genetic background—environmental factors may be the basis of pathophysiological processes (Manolio et al., 2009).

**FIGURE 1:**
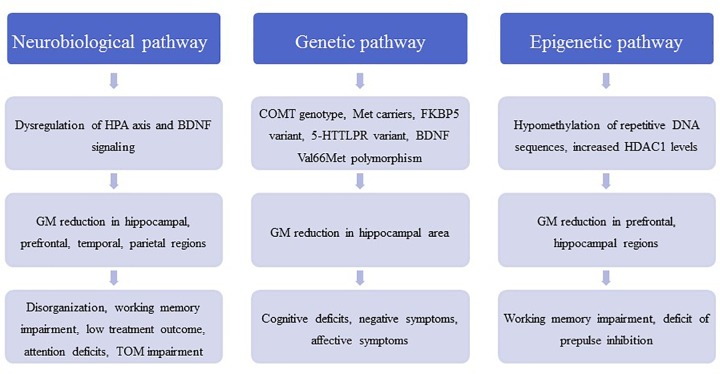
The figure contains a general outline of the three main pathophysiological pathways between childhood trauma and schizophrenic symptoms. These pathways are meant to illustrate the pathological cascade ranging from molecular and (epi-) genetic abnormalities to neuroanatomical changes and eventually to the development of disorder-related symptoms. HPA, hypothalamic–pituitary–adrenal axis; BDNF, brain-derived neurotrophic factor; COMT, catechol-*O*-methyltransferase; FKBP5, FK506 binding protein 5; 5-HTTLPR, serotonin-transporter-linked polymorphic region; HDAC1, histone deacetylase 1; TOM, theory of mind.

Schizophrenia has been regarded as a neurodevelopmental disorder in which defective genes and environmental factors interact and induce symptoms of the disorder. The neurodevelopmental hypothesis proposes that schizophrenia is related to adverse conditions, such as genetic background and environmental factors, which lead to abnormal brain development. Disorder onset and first symptoms occur in early adulthood, after synaptic pruning (Weinberger, 1996; Fatemi and Folsom, 2009). In the two-hit model, a neurodevelopmental disturbance during the perinatal period may lead to dysfunction of neuronal circuits and vulnerability to stress during vulnerable brain periods, and later psychosocial stress or drug abuse, for example, may then trigger the disorder (Schmitt et al., 2014). Today, researchers propose that several hits in the form of genetic and environmental risk factors may interact in a complex way during key periods of neurodevelopment and cumulate in the expression of the disorder state (Figure 1); these risk factors are hypothesized to be common across neuropsychiatric disorders such as schizophrenia, bipolar disorder, and major depression (Davis et al., 2016). The neural diathesis-stress model proposes that psychosocial stress acts upon a pre-existing vulnerability and triggers the symptoms of schizophrenia (Walker and Diforio, 1997). Specific stress factors have been identified that trigger or worsen symptoms of the disorder, such as perceived uncontrollable threats to important goals and socio-evaluative threats (Jones and Fernyhough, 2007). In addition, schizophrenia patients are more emotionally reactive than non-psychiatric controls to stressors such as higher arousal and anxiety (Docherty et al., 2009).

## Neurobiological Effects of Stress

Stress sensitization may play a role in schizophrenia by lowering the vulnerability threshold for the disorder. The neurobiological consequence of stress sensitization involves dysregulation of the hypothalamus-pituitary-adrenal (HPA) axis, which is the major stress neuroendocrine system of the body and is involved in the production of the stress hormone cortisol by the adrenal glands (Holtzman et al., 2013; Schmitt et al., 2014). A stress-induced activation of the HPA axis contributes to dopamine sensitization in mesolimbic areas and increases stress-induced striatal dopamine release (van Winkel et al., 2008). These effects are important because increased dopamine neurotransmission with overstimulation of the D_2_ receptors in several brain regions has been hypothesized in the pathophysiology of schizophrenia, a hypothesis that is supported by the antipsychotic effects of dopamine receptor antagonists (Falkai et al., 2011).

In animal models, acute or chronic stress decreased brain-derived neurotrophic factor (BDNF) levels in the hippocampus, which is involved in synaptogenesis (Neto et al., 2011). In accordance with these findings, stress was found to reduce hippocampal dendrites (Magarinos et al., 2011). Additionally, application of glucocorticoids reduced hippocampal BDNF levels, mimicking the stress reaction (Neto et al., 2011). Animal models have shown that chronic stress or repeated administration of glucocorticoids results in degeneration of hippocampal neurons, with decreased soma size and atrophy of dendrites (Sapolsky et al., 1990; Watanabe et al., 1992). This stress-induced glucocorticoid neurotoxicity (Arango et al., 2001; Frodl and O’Keane, 2013) may contribute to a volume loss in vulnerable brain regions such as the hippocampus; hippocampal volume reduction has been reported for schizophrenia even in early disorder stages (Adriano et al., 2012). Moreover, elevated glucocorticoids can suppress myelination and may affect calcium channels (Damsted et al., 2011). Both mechanisms are related to the pathophysiology of schizophrenia and result in impaired macro- and microconnectivity (Berger and Bartsch, 2014; Cassoli et al., 2015).

In rodents, juvenile social isolation and maternal separation are models of childhood stress, and these animal models have shown lasting effects on the HPA axis along with deficits in hippocampus-dependent learning and memory (Bremne and Vermetten, 2001). The mouse model of juvenile social isolation builds on social isolation immediately after weaning: social isolation leads to schizophrenia-related behavior, such as deficits in prepulse inhibition of the acoustic startle response (PPI) and working memory and decreased social exploration. Furthermore, deficits in oligodendrocyte morphology, reduced myelin thickness, and decreased myelin basic protein and myelin-associated glycoprotein expression have been detected in brain regions (Varty et al., 2006; Makinodan et al., 2012) and resemble the deficit of myelination and oligodendrocytes in schizophrenia (Cassoli et al., 2015). Importantly, in contrast with the effects of adult social isolation this early-induced phenotype cannot be rescued by later social re-integration (Makinodan et al., 2012), implicating impaired recovery, such as in schizophrenia (Table 1).

**Table 1:** Major animal models of early life stress corresponding to childhood trauma.

Study	Stress paradigm	Effects on behavior	Effects on brain biology
Bahari-Javan et al., 2017	Maternal separation	Short-term memory↓Prepulse inhibition↓Novel object recognition learning↓HDAC inhibitor MS-274 rescues ELS induced impairment in PPI and improves novel object recognition learning	Hdac1 mRNA↑HDAC1 protein↑DNA-methylation of the Hdac1 gene at the glucocorticoid receptor (GR-) binding site↓
Makinodan et al., 2012	Juvenile social isolation	Social interaction↓Working memory↓No reversed behavior by reintroduction to a social environment	Oligodendrocytes with simpler morphologyMyelin Basic Protein mRNA↓Myelin Associated Glycoprotein mRNA↓Myelin thickness↓Neuregulin1 type III mRNA↓
Varty et al., 2006	Isolation rearing	Prepulse inhibition↓	


## Stress Response in Patients With Schizophrenia and Individuals at Ultra-High Risk of Developing Psychosis

An increased release of glucocorticoids has been proposed to play a role in the pathophysiology of schizophrenia (Corcoran et al., 2003), and the stress-diathesis model proposes that schizophrenia is associated with elevated baseline and challenge-induced HPA activity (Walker et al., 2008). In addition, cortisol treatment can induce psychotic symptoms (Walker et al., 2008). This model is supported by reports of increased levels of blood cortisol (Ryan et al., 2004) and a blunted cortisol response to stress (Mondelli et al., 2010); the latter was suggested to reflect impaired responsiveness of a desensitized system. Indeed, a meta-analysis on stress-moderating effects of baseline cortisol levels revealed that schizophrenia patients have lower cortisol levels than controls during anticipation of social stress and after exposure to it (Ciufolini et al., 2014). A reduced ability of these patients to appropriately contextualize past experiences has been hypothesized to underlie the missing cortisol response in these experiments (Ciufolini et al., 2014). A blunted cortisol stress reactivity in schizophrenia patients was again reported in a more recent meta-analysis by Zorn et al. (2017), who also pointed toward a possible publication bias as well as an overall small number of studies with properly standardized cortisol protocols as limiting factors for the interpretation of these findings. Moreover, treatment with antipsychotics may have influenced the results (Walker et al., 2008). However, the HPA axis response was also impaired in medication-naïve patients with first-episode schizophrenia, i.e., the cortisol response was flattened, indicating impairments in stress processing (van Venrooij et al., 2012).

According to the neurodevelopmental hypothesis, prodromal and psychotic symptoms occur for the first time in adolescence. In adolescents at ultra-high risk of psychosis (UHR), increased resting cortisol levels have been reported and associated with higher rates of critical statements from relatives and negative self-concept (Carol and Mittal, 2015), indicating that a dysfunction of the HPA axis is related to environmental characteristics. The cortisol level after awakening, which reflects HPA regulation, was also increased in this patient group compared with healthy controls (Nordholm et al., 2018). Additionally, in UHR adolescents a reduced stress responsivity of the HPA axis was correlated with smaller gray matter volumes of the hippocampus and prefrontal, temporal, and parietal cortices, which may represent the neural components in the stress vulnerability model (Valli et al., 2016) (Figure 1). Interestingly, those individuals who subsequently developed psychosis showed a significant blunting of the HPA stress response (Valli et al., 2016).

## Effects of Childhood Maltreatment on Epigenetic Processes

In addition to effects on the hormone system, environmental factors, such as childhood trauma, may contribute to genome–environment interactions; these interactions are mediated by epigenetic processes, such as DNA methylation and histone modifications (Fischer, 2014). Hypomethylation of DNA repetitive sequences has been detected in first-episode schizophrenia patients with a history of childhood trauma (Misiak et al., 2015). Inhibitors of histone deacetylases (HDAC) have been suggested to improve cognitive function and ameliorate disorder pathogenesis in neuropsychiatric disorders such as schizophrenia (Nestler et al., 2016). In schizophrenia patients, we found that the experience of childhood trauma was related to increased HDAC1 levels in blood samples (Bahari-Javan et al., 2017). This corresponds with recent findings that HDAC1 levels are increased in the prefrontal cortex and hippocampus of patients with schizophrenia (Benes et al., 2007; Sharma et al., 2008; Bahari-Javan et al., 2017) (Figure 1). Interestingly, in mice with early life stress as a model of childhood trauma HDAC1 expression is increased in the prefrontal cortex and hippocampus, and these mice display schizophrenia-like behavioral phenotypes, such as deficits in PPI, working memory, and synaptic plasticity (Bahari-Javan et al., 2017) (Table 1). The effects of childhood trauma on epigenetic mechanisms and the relationship with cognition and disorder symptoms should be investigated in more detail in larger studies in schizophrenia patients.

## Childhood Trauma in Schizophrenia: Evidence From Human Studies

Childhood trauma can be assumed to be a severe form of stress that renders individuals more vulnerable to developing schizophrenia. In a meta-analysis of 18 case-control studies (including 2048 patients with psychosis and 1856 non-psychiatric controls), 10 prospective studies (including 41,803 participants), and 8 population-based cross-sectional studies (35,546 participants), Varese et al. (2012) found that adverse experiences in childhood significantly increased the risk to develop psychosis and schizophrenia. The group showed a significant association between childhood adversity, including trauma, and psychosis: the odds ratio was between 2.72 and 2.99, indicating a strong association between childhood adversity and psychosis, including schizophrenia. Epidemiological studies show that exposure to early stress in the form of abuse and neglect in childhood increases the risk to later develop schizophrenia (Bonoldi et al., 2013). In schizophrenia patients, the most frequent subtype of trauma was emotional neglect, but rates of physical abuse and physical neglect were also significantly increased (Larsson et al., 2013). Childhood abuse and neglect are known to have a negative influence on cognition in patients with schizophrenia and bipolar disorder (Shannon et al., 2011). In first-episode schizophrenia patients, exposure to childhood neglect was a predictor for impairment in social cognition and poorer verbal learning, whereas abuse was not (Kilian et al., 2017). A study in Chinese patients with schizophrenia reported that physical neglect was negatively correlated with delayed memory and attention and with the total cognition score (Li et al., 2017). A large study assessed 406 patients with schizophrenia spectrum disorders with the Childhood Trauma Questionnaire and found that physical abuse, sexual abuse, and physical neglect were significantly associated with reduced scores in working memory, executive function, and verbal tasks (Aas et al., 2012b). In another study, metacognitive capacity was lower in patients with childhood emotional abuse (Aydin et al., 2016). Female patients who reported childhood physical abuse had more psychotic and depressive symptoms than both women without this history and men with or without a trauma history (Kelly et al., 2016).

UHR individuals more frequently had a history of childhood trauma, such as emotional and sexual abuse as well as emotional and physical neglect, while emotional neglect in particular was associated with paranoid symptoms (Appiah-Kusi et al., 2017). Even in UHR individuals, a history of childhood maltreatment predicted poorer functioning at follow-up in both those who had transitioned to psychosis and those who had not (Yung et al., 2015). Childhood trauma did not predict transition to psychosis, but after a 2-year follow-up UHR individuals with higher levels of childhood trauma had higher levels of attenuated positive symptoms, general symptoms, and depressive symptoms and lower levels of global functioning (Kraan et al., 2015). In children born to parents with major psychoses, those who were exposed to abuse or neglect had lower IQ and GAF scores and displayed poorer cognitive performance in visual episodic memory end executive functions (Berthelot et al., 2015).

## Interaction of Childhood Trauma With Genetic Factors

Gene–environment interactions have been suggested to play a role in the pathophysiology of schizophrenia (Figure 1). In 429 patients with schizophrenia or schizoaffective disorder, the catechol-*O*-methyltransferase (COMT) genotype moderated the effects of childhood trauma on cognition and symptoms in methionine (met) carriers with a history of childhood physical abuse and more severe positive symptoms; Met carriers with a history of emotional neglect had more severe negative symptoms (Green et al., 2014). In another study, a variant of the FK506 binding protein 5 (FKBP5) gene interacted with childhood trauma and affected attention in both schizophrenia patients and healthy controls (Green et al., 2015b). In patients with schizophrenia and affective disorders, an interaction between a variant in the serotonin transporter gene 5-HTTLPR and childhood trauma was observed in the California Verbal Learning Test (Aas et al., 2012a). A variant of BDNF Val66Met polymorphism was shown to moderate the impact of childhood adversity on later expression of affective symptoms in schizophrenia patients (Sahu et al., 2016). In 249 patients with schizophrenia spectrum disorder, carriers of the met allele of the BDNF gene exposed to high levels of childhood physical and emotional abuse demonstrated poorer cognitive functioning than monozygotic valine carriers. Moreover, Met carriers exposed to childhood sexual abuse showed reduced right hippocampus volume (Aas et al., 2013), suggesting negative effects on neuroplasticity in the brain. On an epigenetic level, a recent review concluded that childhood trauma was associated with global DNA hypomethylation and reduced BDNF gene-expression in first-episode psychosis subjects (Tomassi and Tosato, 2017). However, the literature on gene–environment relationship in the etiology of psychosis is rather heterogeneous as the results from candidate gene studies could quite frequently not be replicated (Zwicker et al., 2018). Thus, epidemiological studies investigating the interplay between familial and environmental factors in the development of psychosis within large cohorts are another valuable resource for further insight. Using these epidemiological approaches, it was found that environmental risk factors, such as childhood adversity, and a family history of affective and psychotic disorders additively impact the psychosis risk across a multidimensional spectrum of positive, negative, cognitive and affective symptoms (Binbay et al., 2012; Pries et al., 2018; Radhakrishnan et al., 2018). Moreover, studies repeatedly showed that childhood adversity and familial liability increased the risk predominantly for positive symptoms of psychosis, such as delusions and hallucinations, as well as affective symptoms (Jeppesen et al., 2015; Smeets et al., 2015; Veling et al., 2016). Therefore, the connection between childhood trauma, familial liability and the onset of psychosis is increasingly being labeled as one of the key mechanisms of the proposed “affective pathway” to psychosis (Isvoranu et al., 2017).

## Brain Structural and Functional Correlates of Childhood Trauma

Emotional neglect in patients with schizophrenia was negatively associated with total gray matter volume and specifically with the density and volume of the dorsolateral prefrontal cortex, which in turn predicted disorganization (Cancel et al., 2015). Interestingly, additive effects of childhood trauma and being a BDNF met carrier on volume loss in the hippocampal subregions cornu ammonis (CA)4/dentate gyrus and CA2/3 have been reported in schizophrenia (Aas et al., 2014). Childhood maltreatment has been associated with reduced hippocampal volume as well as amygdala hyperreactivity and was shown to predict poor treatment outcome (Teicher and Samson, 2013). A functional magnetic resonance imaging study showed that childhood trauma exposure resulted in aberrant function of parietal areas involved in working memory and of visual cortical areas involved in attention. On the basis of these data, the authors hypothesized that childhood trauma in psychosis contributes to alterations in attention during performance of working memory tasks (Quide et al., 2017a). During a theory-of-mind task that reflected social cognition, childhood trauma was associated with activation of the posterior cingulate gyrus, precuneus, and dorsomedial prefrontal cortex in patients with schizophrenia (Quide et al., 2017b). In addition, decreased connectivity between the posterior cingulate/precuneus region and the amygdala was shown in patients with high levels of physical neglect and sexual abuse during childhood (Cancel et al., 2017) (Figure 1). Finally, an fMRI study showed an increased brain response to emotionally negative faces compared with the response to positive faces in patients with psychosis and high childhood trauma, as assessed by the Childhood Trauma questionnaire (Aas et al., 2017). Overall, findings from MRI, genetic, and large-scale gene expression and epigenetic studies often were not reproducible and need to be replicated in larger samples before final conclusions can be drawn.

## Lack of Specificity of Findings for Schizophrenia

It must be noted that effects of childhood trauma are not specific for schizophrenia. In childhood-maltreatment related post-traumatic stress disorder (PTSD), a recent meta-analysis clearly showed bilateral reduction of hippocampal and amygdala volumes in the PTSD group compared to healthy controls (Ahmed-Leitao et al., 2016). In addition, cognitive deficits in different domains such as general intelligence, language, information processing, learning and memory and executive skills have been observed in trauma-exposed children with PTSD compared to controls. Trauma-exposed children with PTSD had poorer general intelligence and visuospatial skills compared with trauma-exposed children who did not develop PTSD (Malarbi et al., 2017). Dysfunction of the HPA axis in PTSD has been reported, particularly hypersensitivity of the glucocorticoid receptor (GR). Single-nucleotide polymorphisms (SNPs) in the GR and FKBP5 gene were associated with PTSD risk and the FKBP5 gene SNP interacted with childhood adversity to moderate PTSD risk (Binder et al., 2008; Castro-Vale et al., 2016). Other neurochemical markers for PTSD include neurotrophic factors such as BDNF (Bandelow et al., 2017). Regarding epigenetic factors, DNA methylation is so far the best studied in PTSD and could be responsible for long-lasting effects of gene–environmental interactions (Rampp et al., 2014). Furthermore, effects of parental trauma could be transmitted to the next generation by epigenetic marks (Ramo-Fernandez et al., 2015).

A meta-analysis showed that childhood psychological abuse and neglect were strongly associated with depression (Infurna et al., 2016). Other factors of childhood maltreatment related to adult depression were emotional abuse, sexual abuse, domestic violence and physical abuse (Mandelli et al., 2015). Regarding genetic factors, the corticotropin-releasing hormone receptor 1 (CRHR1) gene may moderate the effects of childhood trauma on depression (Heim et al., 2009; Ressler et al., 2010). BDNF gene methylation level was correlated with depression (Chen et al., 2017).

## Tools to Assess Childhood Trauma

In the field of childhood trauma research, it is not uncommon to investigate early stress by clinically assessing whether some form of maltreatment took place in the individual’s childhood without applying standardized trauma or maltreatment questionnaires (Choi and Sikkema, 2016; Green et al., 2017). However, in the context of clinical studies and to further both the validity and the reliability of the observed effects in childhood trauma studies, standardized instruments should be used. Below, we present a representative selection of the most commonly used questionnaires because it would be beyond the scope of this article to include all the available ones.

Overall, questionnaires on childhood trauma can be categorized into instruments to diagnose PTSD and more specialized assessment tools, whose goal is to assess childhood maltreatment in depth rather than to validate a DSM or ICD diagnosis. A vast number of PTSD-specific questionnaires are available, but we will give an overview of three structured interviews and one self-report measure. The Structured Clinical Interview for DSM-IV (SCID) and the Composite International Diagnostic Interview (CIDI) are structured interviews that cover the entire spectrum of mental disorders and can be applied by both trained professionals and trained lay interviewers. Both interviews have a specific section on PTSD, are frequently used in epidemiological studies and can be used to validate a suspected diagnosis of PTSD (Kessler et al., 2007; Stein et al., 2014; Guina et al., 2016). Another instrument that has also been extensively reviewed and is regarded by some as the gold standard in diagnosing PTSD is the Clinician-Administered PTSD Scale (CAPS). The CAPS is a 30-item structured interview that should ideally be administered by clinicians and clinical researchers with a working knowledge of PTSD (Weathers et al., 2001, 2018). In addition to these structured interviews, the PTSD Checklist for DSM-5 (PCL-5), a 20-item self-report measure that assesses the 20 DSM-5 symptoms of PTSD, can be used to solidify a PTSD diagnosis (Franklin et al., 2018).

Besides these PTSD-specific diagnostic instruments, a large group of questionnaires focuses on distinct types of childhood maltreatment that do not automatically have to fulfill the PTSD criteria. Childhood maltreatment is usually assessed along the domains of abuse (physical, sexual, emotional/psychological) and neglect (emotional/psychological, physical) (Hovdestad et al., 2015). The most commonly used childhood maltreatment self-reports and semi-structured interviews are described here. The Childhood Trauma Questionnaire (CTQ, Bernstein et al., 1997) is one of the most frequently used self-reports in the current literature (Viola et al., 2016). It has a total of 28 items and measures the above mentioned five types of maltreatment, i.e., emotional, physical, and sexual abuse, and emotional and physical neglect. It also includes a three-item minimization/denial scale to assess the potential underreporting of maltreatment. Another common self-report tool is the Personal Safety Questionnaire (PSQ), which is based on the Conflicts Tactics Scales (Straus and Douglas, 2004). The PSQ queries the occurrence of specific incidents and mainly focuses on physical or sexual abuse; it can be used to sequentially assess incidents that occur in childhood, adolescence, or adulthood. This feature allows researchers to examine both the type (physical or sexual) and timing of abuse over life periods (Rich-Edwards et al., 2011). A questionnaire that specifically focuses on sexual abuse in childhood is the Child Sexual Assaults Scale (CSAS, Koss et al., 1987). This instrument assesses sexual abuse along five subscales: demographic variables subscale, PTSD symptom subscale, center for epidemiologic studies depression subscale, traumatic events questionnaire, and childhood sexual experiences subscale (Yampolsky et al., 2010). An advantage of the CSAS is that it not only assesses possible traumatizing sexual events, but it also checks for PTSD and affective symptoms, therefore mirroring the complex nature of this kind of childhood trauma. Because large multi-center studies have become more important in today’s psychiatry, the Early Trauma Inventory Self Report (ETI-SR) represents a powerful assessment tool that has the advantage of being validated many languages (German, French, Chinese, Spanish, Portuguese, Plaza et al., 2012). The ETI-SR is a 56-item inventory that assesses the presence of childhood trauma with a series of “yes or no” questions and includes specific items for physical (9 items), emotional (7 items), and sexual abuse (15 items) and general trauma (31 items). It also assesses the frequency of trauma, age at trauma, perpetrator, and other variables before age 18 (Bremner et al., 2007; Plaza et al., 2011). The Traumatic Life Events Questionnaire (TLEQ) can be a viable alternative if a broader perspective on possible traumatic or adverse life events is desired. This tool assesses exposure to 16 types of potentially traumatic events, including natural disasters, exposure to warfare, unexpected death of a loved one, severe physical assault, different forms of sexual abuse, and experiences of stalking, and also accounts for the frequency and severity of the named traumatic experiences (Kubany et al., 2000). A rather brief self-report questionnaire is the Adverse Childhood Experiences (ACE) questionnaire (Felitti et al., 1998). In a total of 17 questions, this questionnaire assesses childhood abuse within the domains of psychological, physical, and sexual abuse. Additionally, it sets itself apart from many other self-reports because it also includes four categories of childhood exposure to household dysfunction, i.e., substance abuse, mental illness, violent treatment of mother or stepmother, and criminal behavior in the household.

With regards to semi-structured interviews, two more questionnaires are of interest because they both have specific advantages and can be useful in clinical studies: The Early Trauma Inventory and the Children’s Life Events Scale (CLES). The former instrument is a semi-structured interview that assesses four domains of traumatic experiences (physical, emotional, and sexual abuse and general traumatic experience) and then addresses the most serious trauma in an additional question (Bremner et al., 2000). This additional question can be very useful in clinical settings because of the potential need for an extended conversation about the most burdensome issue. The CLES, which is an expansion of The Source of Stress Inventory (Chandler, 1981), is a checklist composed of 50 moderate-to-major stressful childhood events that covers categories such as negative emotional feedback, family deaths, maltreatment, failure in school, and family dysfunction (Crossfield et al., 2002; Grandin et al., 2007).

When selecting a questionnaire, equally important to the frequency of use is the analysis of the resulting dataset. Therefore, in the next section we critically discuss current analysis methods and give an outlook on advanced mathematical analysis methods.

## Novel Approaches for Assessing and Analyzing Childhood Maltreatment

**FIGURE 2:**
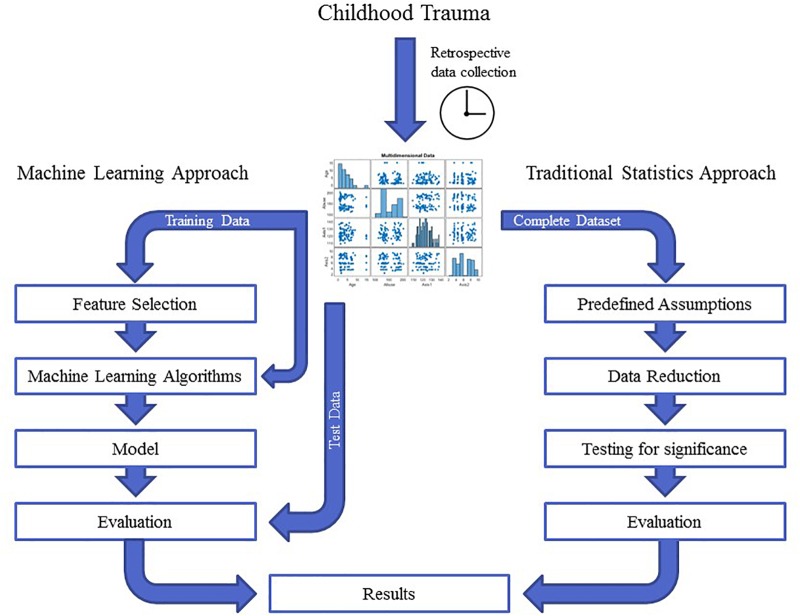
The graph depicts the different workflows in traditional statistics compared with machine learning approaches. In traditional statistics, one approaches a dataset with predefined assumptions, reduces the entire dataset according to those assumptions and then tests a certain hypothesis for significance. In contrast, unbiased machine learning approaches split the dataset into training and test data and let an algorithm learn from the training data in an unbiased and hypothesis-free manner. The evaluation of the analysis then depends on how well the model performs when applied to the test data. These two approaches can yield quite different results.

**FIGURE 3:**
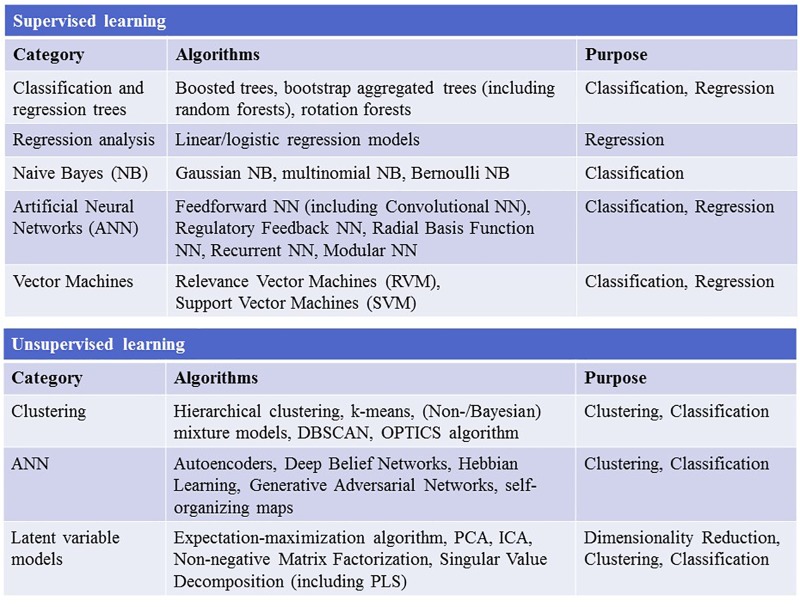
The figure depicts the most widely used supervised and unsupervised learning algorithms. NN, neural network; DBSCAN, Density-Based Spatial Clustering of Applications with Noise; OPTICS, ordering points to identify the clustering structure; PCA, principal component analysis; ICA, independent component analysis; PLS, partial least squares.

Childhood trauma poses several challenges when it comes to data integration and data analysis, mainly with regards to the temporal resolution and the reciprocity and interdependency of the observed phenotypes. The temporal problem arises because most adverse events, which presumably occurred in childhood, can only be assessed retrospectively and are therefore prone to a certain recollection bias (MacDonald et al., 2015). Furthermore, the sequence in which adverse events in childhood were experienced and psychiatric symptoms developed is often unclear. Another issue lies in the reductionist steps that most studies take during “preprocessing” of the data on adverse experiences or events. In the first step, the data are categorized into specific overarching domains, such as physical or emotional abuse (Morgan and Fisher, 2007), which removes a great amount of the detailed information given by the individual. The next quite common reductionist step is to build sum scores for these domains or, in some cases, a total score for all domains (Hovdestad et al., 2015). In this second step, information given by patients is summarized into nominal or ordinal categories, for example “childhood trauma present” versus “childhood trauma absent” or “high childhood trauma,” “medium childhood trauma,” and “low childhood trauma” (Daruy-Filho et al., 2011; Agnew-Blais and Danese, 2016). This step removes a great amount of variance and heterogeneity within the dataset that could be important for future analyses. Overall, these preprocessing steps take the interdependency and reciprocity of these adversary factors and their association with the observed psychopathology out of the equation. The possible interactive effects between various kinds of adverse experiences, psychopathological symptoms, and organic features (i.e., structural and functional MRI, DNA variants, gene expression, or epigenetic mechanisms) of the affected individual are largely removed. Thus, most current studies in the field of childhood trauma research are trying to investigate a highly dynamic phenomenon, in which various risk and protective factors interact with each other and produce complex clinical and organic phenotypes, with simplified models that use ordinal and nominal grouping and univariate statistics (Figure 2). At the same time, age and sex are mostly controlled for, even though age- and sex-specific effects are found in various psychiatric disorders (Cascio et al., 2012; Gur and Gur, 2016). Based on these methodological issues, the potential advantage of using unbiased and explorative machine learning and multivariate analysis techniques becomes evident (Dwyer et al., 2018b; Jollans and Whelan, 2018). While supervised learning algorithms such as neural networks, tree-based algorithms and vector machines can deliver insights into psychiatric disorders through classification and regression of labeled training data (Bzdok et al., 2018), unsupervised learning algorithms are able to complement this by uncovering latent structures within an unlabeled training dataset (Figure 3). Hence, latent variable models based on factor analysis or singular value decomposition (i.e., principal component analysis, non-negative matrix factorization, partial least squares) might be used to explore associative effects between variables of interest (Jessen et al., 2018; Stein-O’Brien et al., 2018). In this context, these associative effects could then be used to further explore causal links between different kinds of childhood adversity, psychopathology, and organic features, e.g., MRI images or DNA expression profiles (Krakauer et al., 2017). Other unsupervised techniques like hierarchical clustering or self-organizing maps could be employed to find mathematically sound clusters of adverse childhood effects or certain phenotypical or organic patterns of childhood trauma, that would be lost if one kept to the overly restrictive approach of using DMS diagnoses or categorical/nominal grouping of childhood trauma loading (Dwyer et al., 2018a). Another interesting topic for analysis with multivariate tools is the timeline of each individual, which is defined by specific childhood trauma experiences and onset and development of certain symptoms.

The effect of childhood trauma on psychopathology and organic variables, such as brain structure and DNA variants (see above), cannot be reduced to a static observation, and we need to consider longitudinal data, the course of disorder, and the biography of each individual. Therefore, mixture models involving (Hidden) Markov Models, Directed Graphical Models, and Bayesian Networks, would help to model, predict and consequently explain the connection and evolution of childhood trauma, psychopathology, and, if desired, its organic correlates (Orphanou et al., 2014; Ryali et al., 2016; Seltman et al., 2016). Some of these approaches have already been undertaken in the field of PTSD research (Galatzer-Levy et al., 2014; Karstoft et al., 2015); however, to our knowledge in the field of childhood trauma and psychosis research no studies have yet been published on machine learning techniques (Figure 1). Therefore, this still unexplored field of unbiased, data-driven childhood trauma research has exciting potential and should be one of the priorities for future research.

## Author Contributions

PF, BM, AS, and DP designed this manuscript. DP, AS, LK, FS, SP, BM, AF, TS, NK, and PF managed the literature searches, interpreted the data, and prepared the manuscript. All authors contributed to and approved the final manuscript and reviewed it critically for important intellectual content.

## Conflict of Interest Statement

PF has been an honorary speaker for AstraZeneca, Bristol Myers Squibb, Lilly, Essex, GE Healthcare, GlaxoSmithKline, Janssen Cilag, Lundbeck, Otsuka, Pfizer, Servier, and Takeda and has been a member of the advisory boards of Janssen-Cilag, AstraZeneca, Lilly, and Lundbeck. AS was honorary speaker for TAD Pharma and Roche and a member of Roche advisory boards. The remaining authors declare that the research was conducted in the absence of any commercial or financial relationships that could be construed as a potential conflict of interest. The handling Editor declared a past co-authorship with one of the authors PF.
